# Effects of a Computer Vision–Based Exercise Application for People With Knee Osteoarthritis: Randomized Controlled Trial

**DOI:** 10.2196/63022

**Published:** 2025-05-12

**Authors:** Dian Zhu, Jianan Zhao, Tong Wu, Beiyao Zhu, Mingxuan Wang, Ting Han

**Affiliations:** 1 School of Design, Shanghai Jiao Tong University, Dong Chuan rd, No 800, Shanghai, 200140, China, 86 18901626266; 2 College of Fashion and Design, Donghua University, Donghua University, Shanghai, China; 3 Department of Plastic and Reconstructive Surgery, Shanghai Jiao Tong University Ninth People's Hospital, Shanghai, China

**Keywords:** knee osteoarthritis, behavior change theory, application, digital health, randomized controlled trial, knee, osteoarthritis, behavior, vision, exercise, app, applications, exercise rehabilitation, rehabilitation, older adults, self-efficacy, physical function

## Abstract

**Background:**

Exercise is a primary recommended treatment for knee osteoarthritis (KOA), as it helps alleviate symptoms and improves joint functionality. Personalized exercise programs, tailored to individual patient needs, have demonstrated promising results in maintaining physical fitness and enhancing overall well-being. In recent years, digital health applications have emerged as innovative tools for supervising and facilitating rehabilitation programs. Leveraging computer vision (CV) technology, these applications offer the potential to provide precise feedback and support personalized exercise interventions for patients with KOA in a scalable and accessible manner.

**Objective:**

This study aims to evaluate the impact of a CV–graded exercise intervention application over a 6-week period on clinical outcomes in patients with KOA . The outcomes were compared to those achieved through conventional exercise education by videos.

**Methods:**

A randomized controlled trial was conducted with 60 participants aged 60‐80 years, recruited through community administrators between July 2023 and September 2023. Participants were randomly assigned to one of two groups: the graded exercise application group (n=32) and the exercise education brochure group (n=28). The primary outcomes assessed were short-term changes in pain, physical function, and stiffness as measured by the Western Ontario and McMaster Universities Arthritis Index (WOMAC). Secondary outcomes included assessments of participants’ affective state, self-efficacy, quality of life, and user experience.

**Results:**

The study recruited 60 participants, including 26 males and 34 females. Analysis revealed statistically significant improvements in physical function (*P*=.02) and self-efficacy (*P*=.04) in the graded exercise application group compared to the exercise education brochure group after the intervention. While improvements in pain and stiffness were observed in both groups, these changes were not statistically significant. In addition, participants in the graded exercise application group reported a positive user experience, highlighting the application’s usability and engagement features as beneficial to their rehabilitation process.

**Conclusions:**

The findings suggest that the CV-based graded exercise intervention application effectively improves physical function and self-efficacy among patients with KOA . This digital tool demonstrates the potential to enhance the quality and personalization of exercise rehabilitation compared to traditional methods. Future studies should explore the application’s long-term efficacy and replicability in larger community-based populations, with a focus on sustained engagement and adherence to rehabilitation programs.

## Introduction

Knee osteoarthritis (KOA) is a common and chronic joint disorder that is growing in prevalence as the world’s population ages [1,2]. Exercise has been acknowledged as a nonpharmacological intervention modality for the treatment and prevention of musculoskeletal disorders, including osteoarthritis, osteoporosis, back pain, and rheumatoid arthritis [3]. Specifically, it has been empirically demonstrated that participating in appropriate physical exercise while being monitored by a physiotherapist effectively maintains physical health and athletic ability [4]. In order to optimize joint flexibility, strengthen muscles, and reduce strain, they develop individualized training plans that are prescribed in accordance with the patient’s condition [5]. Nevertheless, this procedure imposes considerable financial burdens on the patients and requires a significant time commitment from the caregivers [6].

Digital health interventions have the potential to mitigate the time and resource limitations faced by patients with KOA by offering education and self-management through web-based platforms or applications that are scalable, inexpensive, readily available, and high coverage [7-9]. Digital health interventions have been shown to potentially offer benefits in the management of musculoskeletal disorders [10,11]. Nevertheless, there are still several digital intervention tools that demonstrate inconsistent levels of effectiveness. For instance, physical therapy sessions and digital applications were shown to be successful in improving physical function [12], while a program using wearable devices to deliver physical activity counseling was deemed ineffective in promoting physical function [13]. This is attributed to the varying functionalities that are accessible and the absence of support for these functionalities in influencing user behavior.

Research has indicated that the integration of behavior change theory offers the potential for both positive behavior modification and amelioration of negative emotions [14]. Behavior modification approaches are comprised of three essential components: goals and planning, feedback and monitoring, and repetition and replacement [15]. Goals and planning promote increased general physical activity by setting short-term goals and developing evidence-based progressive individualized exercise plans [16]. Monitoring and feedback oversee progress and acquire immediate feedback on the accomplishment of objectives [17]. The application primarily consists of recurring and substituted generalized and graded tasks, which incorporate behavioral exercises and target behaviors [18,19]. A systematic review revealed that exercise programs through positive feedback, effort reinforcement, motivating techniques, and graded interventions had a higher likelihood of adhering to therapeutic exercise [20].

Previous research has established that patients can improve their management of illnesses through the implementation of goal-setting and planning strategies (monitoring, documenting, etc) as well as the consistent practice and substitution of behaviors (performing repetitive movements, engaging in daily timed exercise, etc) [21]. Clinical treatment is facilitated through the utilization of sensor technology, which provides objective monitoring data for noninvasive evaluation of knee function [22,23]. Despite the availability of various feedback programs formulated to assist patients in adhering to exercise regimens, a subset of patients diagnosed with KOA continue to encounter suboptimal exercise outcomes [9,24,25]. Previous reviews have highlighted that one of the primary objectives of CV-based applications is to calculate joint angles, enabling the identification and counting of correct postures during rehabilitation training. In addition, accurately assessing whether exercises are performed correctly is another critical goal, ensuring that the movement sequence aligns with rehabilitation standards [26,27]. Given the requirement for enhanced and immediate visual input to enable the execution of rehabilitation exercises, the application of computer vision (CV)–algorithms for body position tracking appears to hold considerable promise [28,29]. Nevertheless, clinical investigations evaluating most of these CV-based rehabilitation programs have not yet been conducted.

Given the tremendous potential of these applications for rehabilitation exercises, a program based on CV was developed. The application facilitates the development of a graded exercise rehabilitation program for patients and aids them in self-monitoring the program’s implementation. The main objective was to assess the effects of using a CV-graded exercise intervention application (after 6 weeks) on clinical outcomes (pain and physical function) among patients diagnosed with KOA. The secondary outcome was to investigate the effects of application implementation on the affective state and self-efficacy of patients with KOA.

## Methods

### Study Design

This parallel, 2-arm, unbalanced randomized, single-blinded controlled trial was conducted following the CONSORT (Consolidated Standards of Reporting Trials) Checklist 1 [30] and CONSORT-EHEALTH (Electronic and Mobile Health Applications and Online Telemedicine Reporting Trials) [31] guidelines. The trial was carried out from January 2024 to May 2024 in community activity centers in Shanghai. Participants in the intervention group used CV-based exercise assessment and intervention system designed specifically for patients with KOA, while those in the control group used an exercise rehabilitation education video.

However, during the pilot study of 4 participants, it was observed that some of them lacked adequate digital health literacy and were unable to independently configure and operate the experimental devices. To address this issue, the research team modified the experimental protocol, organizing in-person sessions at community centers 3 times per week, where trained personnel facilitated the intervention tasks for participants.

### Participants Recruitment

Participants underwent a 6-week intervention comprising CV-based graded exercise sessions 3 times per week, designed to deliver personalized rehabilitation exercise plans and instructional videos. Recruitment was conducted from July 2023 to January 2024[d1] in Shanghai through invitations extended by community managers and posters distributed in nursing homes.

Inclusion criteria were as follows: (1) age ≥50 years, (2) radiologically confirmed diagnosis (KL grade ≥2, pain in affected joints) in accordance with American College of Rheumatology clinical criteria, (3) mean overall pain severity ≥4-point numeric rating scale (NRS), (4) good communication and comprehension skills without significant cognitive deficits, and (5) the ability to operate an electronic device with some proficiency.

Exclusion criteria were as follows: (1) participants with severe knee pain and discomfort, (2) participants with severe organic lesions of vital organs such as heart, brain, and kidney, and (3) participants who had been undergoing knee arthroplasty.

Only participants who finished the final 6-week assessment of the experiment and did not withdraw were included in this study.

### Interventions

#### Intervention Group (Applications)

This study developed an exercise evaluation and intervention system for individuals suffering from KOA (Multimedia Appendix 1). The objective was to assist participants in preserving and enhancing their knee joint motor function by implementing a tailored progressive rehabilitation regimen and providing immediate feedback. The intervention consists of three components: (1) establishment of a comprehensive knee function assessment system; (2) delivery of secure and precise exercise interventions for participants and offer immediate feedback on the impact of exercise intervention by continuously collecting knee function metrics in real-time; (3) issuing timed tasks to encourage participants to exercise accordingly.

##### Status Assessment

**Figure 1.**
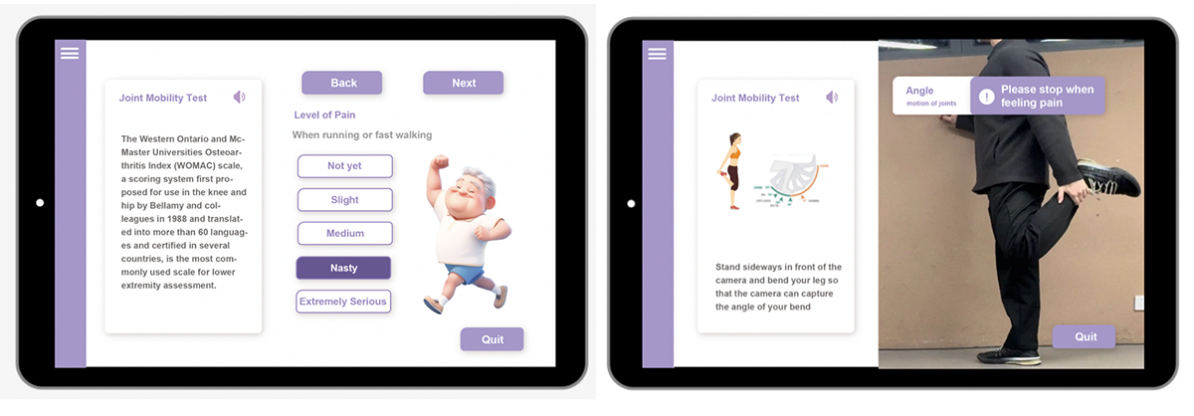
Knee function evaluation system.

A comprehensive knee function assessment system was developed using CV technology, an electronic Western Ontario and McMaster Universities Osteoarthritis Index (WOMAC) questionnaire, joint mobility detection, and mobile gait data monitoring. The tool offers a scientific and all-encompassing evaluation instrument for individuals afflicted with knee joint disorders (Figure 1).

First, a methodical evaluation of the participants’ joint pain, rigidity, and dyskinesia was conducted with the WOMAC scale. Furthermore, CV technology was used to identify and document the frequency and quantity of movements executed by the participants throughout the physical function assessments. A physical function test involves the participants repeatedly performing a designated movement, and physical function is evaluated by tally-marking the number of movements completed within a specified time interval. CV is also used to dynamically gather the utmost angle of knee flexion. The remote gait data tracking feature relies on Apple’s HealthKit, which enables the system to collect gait data over a period of time. This includes measurements such as step length, step frequency, walking speed, bipedal symmetry, and bipedal support duration. However, the user must provide authorization for the system to access this data. Continuous and extended monitoring of this data allows for evaluation of the advancement of the condition and offers participants insights into their walking patterns and behavioral habits.

##### Action Detection

**Figure 2.**
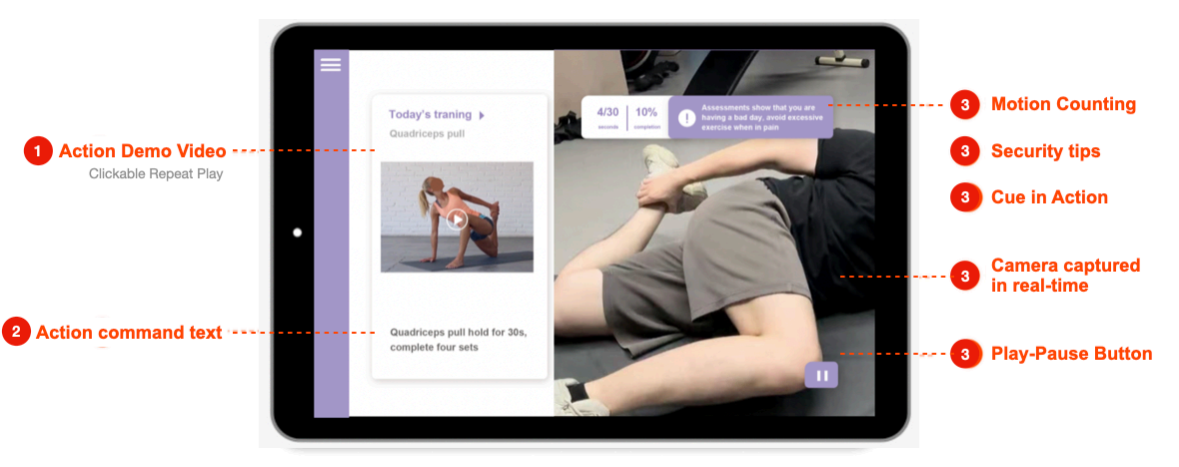
Application of computer vision technology.

Standardizing movements during exercise interventions has an impact on the body’s dynamics during exercise and physical function testing. Hence, aside from the completion status of the action, they also prioritize factors such as the precision and efficacy of the movement (Figure 2).

Through the application of CV technology, the system is able to capture the participants’ movement posture. The system collects data from posture capture to create a real-time analysis of movement completion as the participants conduct the scheduled exercise. This enables immediate feedback to be given to the participants during the training regimen. The screen displays a physical function test demonstration, which the older adults observe and subsequently replicate. The software generates a tally of the older adults’ completed motions within a specified time frame. Throughout the entire procedure, the program is equipped with a function that can assess hazardous scenarios. If a fall is imminently possible, the physical function exercise program will promptly halt and emit an audible alert. Upon completion of the test, the older adults could access and review the results displayed on the screen. By conducting regular physical function assessments, individuals can acquire an impartial comprehension of the condition of their knee joint and obtain an accurate assessment of their physical mobility that is supported by the knee joint.

##### Exercise Rehabilitation

Following the evaluation process, participants are granted the ability to implement a customized exercise regimen that aligns with their rehabilitation strategy and integrates digital evaluation techniques to obtain immediate feedback and suggestions for modifications. The goal is to prioritize the rehabilitation requirements of the participants through the implementation of a customized rehabilitation program and consistent remote care.

The primary purpose of the exercise intervention is to implement an interactive exercise program that captures and assesses the participants’ movements by CV during exercise. The user performs the prescribed movements in a sequential manner in order to achieve the daily workout objectives. The subsequent interface will present the exhibited actions, and older individuals can imitate each movement by following the demonstration video (Table 1). A brief interval is observed between each set of movements, and the exercise routine concludes once the task of the designated movements has been completed. The degree of standardization of the movement is denoted by the range of motion, and feedback pertaining to this degree of standardization is additionally acquired following each movement completion.

**Table 1.** Description of rehabilitation exercise movements.

Action	Action instructions
Calf raises	With this calf raise exercise, calf muscles can be strengthened so that patients can walk and climb stairs with ease.
Lateral raise (leg)	This side leg raise will strengthen the hip abductors and make everyday tasks like getting in and out of the car easier.
Knee lift exercise	Strengthen the muscles around the buttocks with this knee lift exercise.
Seated knee lift	It can be used to improve the ability to get up from a chair, get in and out of bed or a car. Patients can start with the easiest and then slowly progress to the hardest.
Seated rise	Increase the flexibility and strength of the hip extensors with this standing hip extension exercise.
Hip stretches	Maintain range of motion in the legs with heel and toe-tapping exercises.
Heel and toe tapping	Improved balance and coordination through toe-tapping exercises.

##### Remote Follow-Up

The primary functions also encompass the formation of a rehabilitation community and conducting remote follow-up. In the context of rehabilitation, incentives like as competition and leader-boards are implemented to motivate participants to actively engage in remote therapy and foster mutual contact within the rehabilitation community. Another purpose of the remote follow-up feature is to establish remote communication channels with outpatient doctors in order to minimize the time and effort participants spend on medical treatment.

### Control Group (Exercise Rehabilitation Education Video)

Participants in the control group received a video with instructions for daily exercise movements, which were similar to those in the application (Table 1).

### Outcomes

Participants received validated digital questionnaires at baseline, after 6 weeks of intervention, or face-to-face questionnaire completion at offline follow-up. Participants did not receive (financial) incentives or other compensation for completing the questionnaire or the study. Demographic data were collected at baseline and described in detail in the outcome measures section.

### Main Outcomes

Pre and postintervention status was assessed by the WOMAC scale [32]. Respondents reported the severity of their usual arthritis pain from 0 (none) to 20 (severe), functional impairment due to osteoarthritis from 0 (none) to 68 (severe), and knee stiffness from 0 (none) to 8 (severe).

### Secondary Outcomes

#### The Arthritis Self-Efficacy Scale

The Arthritis Self-Efficacy Scale (ASES-8) assesses participants’ confidence in their ability to manage arthritis pain and its impact on function [33,34]. Scores range from 1 (very uncertain) to 8 (very certain). Responses are averaged, with higher scores indicating higher self-efficacy.

#### Geriatric Depression Scale (GDS)

Negative emotions were measured by the Geriatric Depression Scale (GDS), whose 30 entries represent the symptoms of depression in old age and contain low mood, decreased activity, irritability, withdrawal from painful thoughts, and negative appraisal of the past, present, and future [35]. Scores range from 0 (none) to 30 (extremely). Higher scores indicate greater positive or negative affect.

#### Quality of Life

Quality of Life (AQoL-6D [Assessment of Quality of Life-6D] version) ranges from -0.04 to 1.00, with higher scores indicating better quality of life [34].

#### Range of motion

Range of motion [36], consisting of both extension (ie, the ability to straighten the leg) and flexion (ie, the ability to bend the leg), with scores ranging from 0 to 135 (higher scores indicate better joint mobility).

#### User Experience Questionnaire

The User Experience Questionnaire (UEQ), which is used to quickly assess the user experience of an interactive product, includes the traditional metrics of ease-of-use aspects: efficiency, comprehensibility, trustworthiness, attractiveness, motivation, and freshness [37].

### Sample Size

The sample size for this study was determined using a “Sample Size Calculation for Two-Group Mean Comparison.” Considering the characteristics of the intervention and control groups (digital application group and education group, respectively), the calculation was informed by the parameters reported in previous studies [38,39].

The sample size calculation was conducted assuming a difference between the intervention and control groups (μ₁-μ₂) of 8.8 (SD 9) Using a 2-sided *t* test with a significance level (*α*) of .05 and a statistical power (1-*β*) of 80%, the minimum required sample size was estimated to be 36 participants, with 18 participants in each group. This ensured adequate power to detect meaningful differences between the groups.

Based on previous experience from our research team’s extensive work in community-based digital rehabilitation, we have observed a high dropout rate among older adults in such settings. To address this, we implemented over-recruitment, increasing the sample size by at least 50% beyond the calculated minimum to ensure sufficient statistical power for final analyses.

### Randomization, Allocation Concealment, and Blinding

The randomization process will involve stratifying the 60 participants into 15 blocks, each containing 4 participants. Within each block, participants will be randomly allocated to either the intervention group or the control group (received the exercise rehabilitation education video). Experienced statisticians used SAS (SAS Institute) for randomization grouping to ascertain that the random assignment of researchers was concealed for treatment assignment. Due to the nature of the study, participants were not blinded as they knew whether they received the program during the intervention period, while the statisticians were blinded to the group assignment.

Following the assignment, individuals in the intervention group were sent an email by the researcher (JZ) containing instructions on how to access the computerized visual grading exercise application and details regarding the field trial arrangements. Participants in the control group were notified via email about their assignment and received an exercise rehabilitation education video. Throughout the trial, participants were provided with the option to contact the researchers by phone or email if they had any inquiries regarding the application or the study.

### Statistical Methods

The analyses were performed by statisticians (MW and TW) on SPSS 26.0 (IBM) software and following the intention-to-treat principle. Baseline characteristics of participants who provided and did not provide the primary outcome were compared using *t* tests or *Χ*^2^ tests. The Mann-Whitney *U* test was employed to examine between-group and within-group data pre and post experiment. For this work, a significance level (*α*) of .05 was established for all statistical analyses. Differences were deemed statistically significant for the purposes of this study if the *P* value was less than .05.

### Ethical Considerations

The clinical trial was registered (NCT06220565) and approved by the Ethics Committee of Shanghai Jiao Tong University (H20240039I). All participants were required to complete an informed consent form and were informed that their data would remain anonymous. After the completion of the experiment, each participant will receive a US $50 financial compensation.

## Results

### Baseline

**Figure 3.**
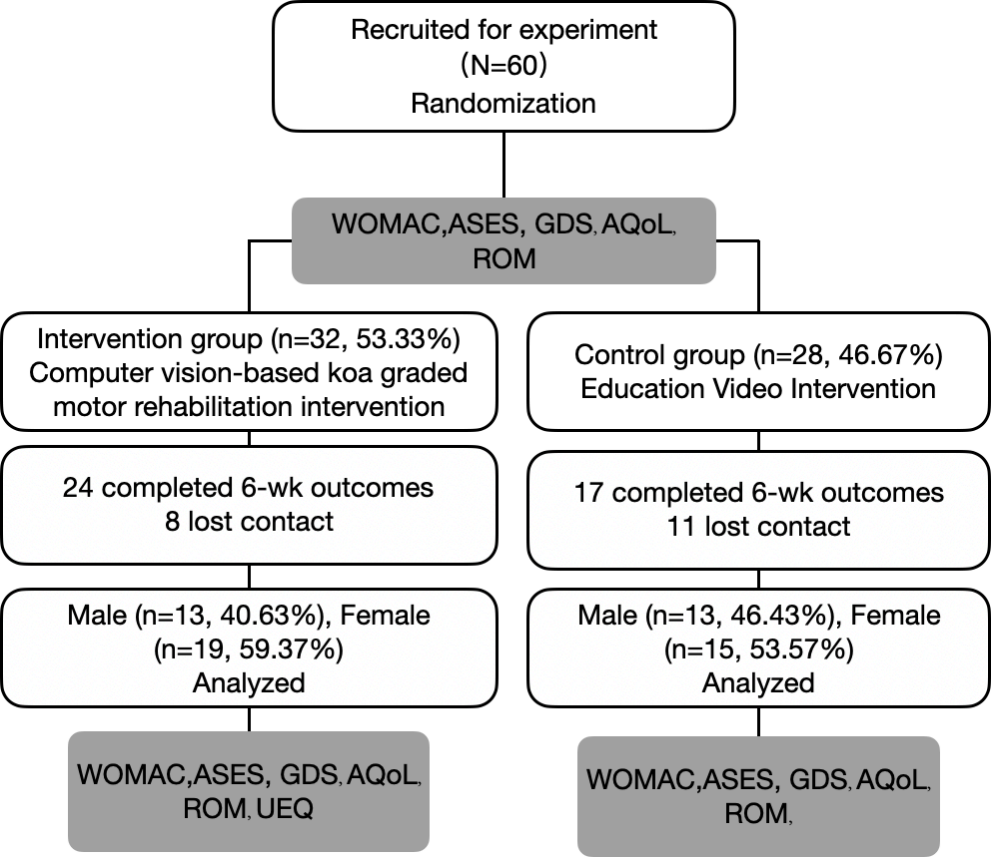
The flowchart of the experiment. AQol: Assessment of Quality of Life; ASES: Arthritis Self‐Efficacy Scale; GDS: Geriatric Depression Scale; ROM: range of motion; UEQ: User Experience Questionnaire; WOMAC: Western Ontario and McMaster Universities Arthritis Index.

Between June and September 2023, a randomization process involving a total of 60 people was conducted who fulfilled the recruiting criteria (32 in the application group and 28 in the educational video group) (Figure 3). A total of 41 (68.33%) participants successfully fulfilled the major outcome measure after 6 weeks, including 24/32 (75%) participants from the application group and 17/28 (60.7%) participants from the instructional video group. The average (SD) age of the sample was 68.8 (SD 6.4), and most of the sample was female (37/60, 61.67%). There were no significant differences between the groups at baseline (Table 2).

Initially, with the exception of 2 individuals, all participants maintained the belief that the rehabilitation application would aid them in the more precise rehabilitation of their motions. In addition, 87% (135/155) of participants believed that the application would aid them in recovering physical function more effectively. A considerable percentage indicated that they were not presently engaged in active therapy, despite experiencing knee discomfort. There were very few negative incidents reported, with only one instance reporting a negative incident.

**Table 2.** Baseline characteristics of participants.

Outcome	Intervention (n=32）	Control (n=28)	All (N=60）	*P* value
Age (years), mean (SD)	68.59 (7.16)	67.64 (7.85)	68.15 (7.28)	.82
Female, n (%）	19 (60)	15 (54)	35 (57)	.79
Medication, n (%）				
Yes	11 (34)	10 (36)	21 (35)	.43
No	21 (66)	18 (64)	39 (65)	
Symptom duration (years), n (%）				
<2	7 (22)	6 (23)	13 (22)	.98
2‐10	21 (66)	18 (63)	39 (65)	
>10	4 (12)	4 (14)	8 (13)	
Primary outcome (WOMAC)^a^, mean (SD)				
Physical function	24.13 (2.7)	25.39 (2.99)	24.72 (2.9)	.12
Pain	8.87 (1.59)	8.64 (1.52)	8.76 (1.57)	.98
Symptoms	3.47 (1.19)	3.39 (1)	3.43 (1.1)	.9
Secondary outcome, mean (SD)				
ASES-8^b^	49.97 (7.49)	48.32 (7.25)	49.97 (7.37)	.41
GDS^c^	6.28 (1.84)	6.32 (0.08)	6.30 (1.8)	.9
AQoL-6D^d^	0.65 (0.1)	0.58 (0.08)	0.62 (0.09)	.06
ROM^e^	104.75 (7.16)	103.93 (7.93)	104.37 (7.48)	.57

a WOMAC: Western Ontario and McMaster Universities Arthritis Index.

b ASES-8: Arthritis Self-Efficacy Scale–8.

c GDS: Geriatric Depression Scale.

d AQoL-6D: 6-Dimensional Assessment of Quality of Life scale.

e ROM: range of motion.

### Primary Outcomes

**Figure 4.**
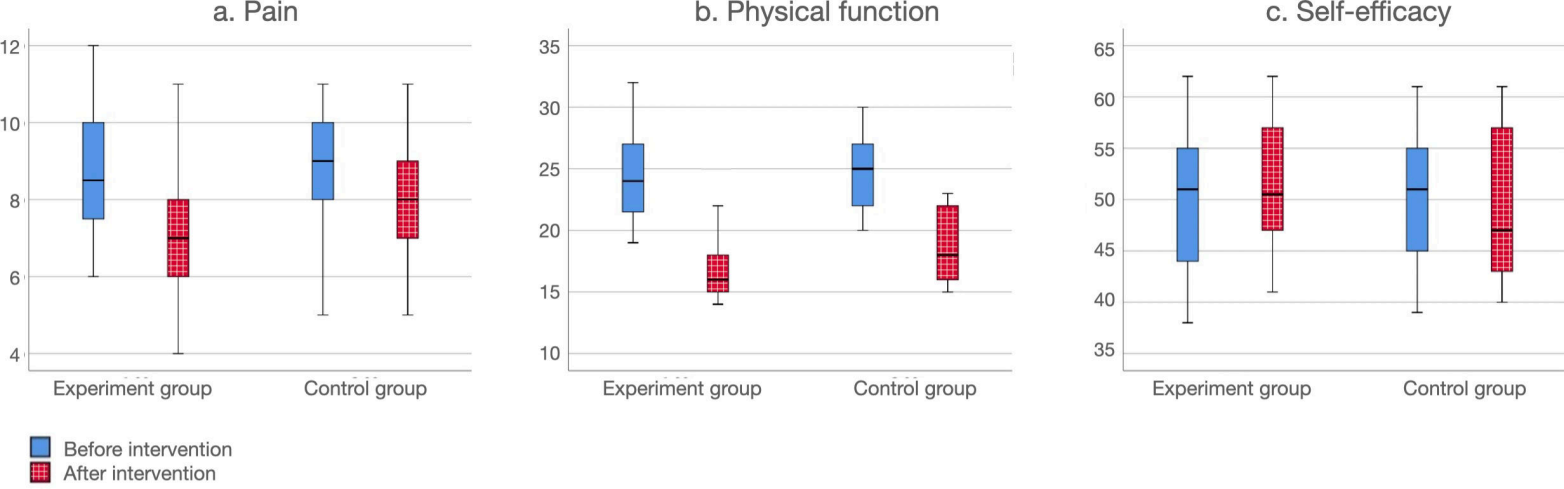
Box plots illustrating pre and postintervention comparisons of the outcomes.

Table 3 and Figure 4 describe the application program in the intervention and control groups. At week 6, the application group demonstrated improvement in physical activity impairment (*P*=.02) compared to the control group, and 15/24 participants reported subsequent adherence to home exercise or community fitness. Although there was a significant within-group difference in the intervention group before and after the intervention (*P*<.001), there was no evidence of a significant improvement in pain (*P*=.96) between the intervention group and the control group. In addition, there were no within-group (*P*=.21) or between-group (*P*=.49) differences in stiffness before or after the intervention group experiment.

**Table 3.** Primary and secondary intervention outcomes.

	Intervention group (n=24）			Within group	Control group (n=17）			Within group	Between group
	Baseline	6-wk	Difference (95% CI)	*P* value	Baseline	6-wk	Difference 95% CI	*P* value	*P* value
Primary outcomes (WOMAC)^a^									
Physical function	24.17 (3.24)	17.08 (2.7)	−7.08 (−9.98 to 4.18)	.00^b^	24.76 (3.27)	18.71 (2.99)	−6.06 (−7.97 to −4.15)	.42^c^	.02^c^
Pain	8.83 (1.79)	7.21 (1.58)	−1.63 (−3.38 to 0.06)	.00^b^	8.94 (1.82)	7.76 (1.52)	−1.18 (−3.3 to 0.94)	.00^b^	.96
Symptoms	3.5 (1.25)	3.25 (1.19)	−0.25 (−1.19 to 0.69)	.21	3.41 (1)	3.35 (0.99)	−0.06 (−0.81 to 0.69)	.74	.49
Secondary outcomes, mean (SD)									
ASES-8^d^	49.79 (6.96)	51.75 (6.54)	1.96 (-2.3 to 6.22)	.003^c^	48.32 (7.25)	49.47 (7.37)	−0.47 (−4.44 to 3.5)	.79	.04^c^
GDS^e^	6.33 (1.86)	5.58 (1.83)	−0.75 (−3.06 to 1.56)	.13	6.24 (1.3)	5.65 (1.8)	−0.59 (−2.9 to 1.72)	.42	.98
AQoL^f^	0.45 (0.18)	0.48 (0.19)	0.05 (−0.09 to 0.19)	.08	0.55 (0.19)	0.52 (0.21)	−0.03 (−0.23 to 0.17)	.56	.09
ROM^g^	104.63 (7.72)	104.96 (7.79)	0.33 (−8.58 to 9.24)	.76	103.29 (7.82)	104.23 (9.52)	0.94 (−8.62 to 8.62)	.59	.58

a WOMAC: Western Ontario and McMaster Universities Arthritis Index.

b P<.001.

c P<0.05.

d ASES-8: Arthritis Self-Efficacy Scale–8.

e GDS: Geriatric Depression Scale.

f AQoL: Assessment of Quality of Life.

g ROM: range of motion.

### Secondary Outcomes

The secondary outcome data showed a significant difference between the 2 groups in terms of the change in self-efficacy after 6 weeks (*P*=.04). Nevertheless, the study found no evidence to suggest that the application program intervention led to a noteworthy enhancement in geriatric depression (*P*=.93) and range of motion (*P*=.58) as compared to the control group. The intervention group showed a slight improvement in quality of life. However, this improvement was not statistically significant overall (*P*=.09).

### User Experience

**Figure 5.**
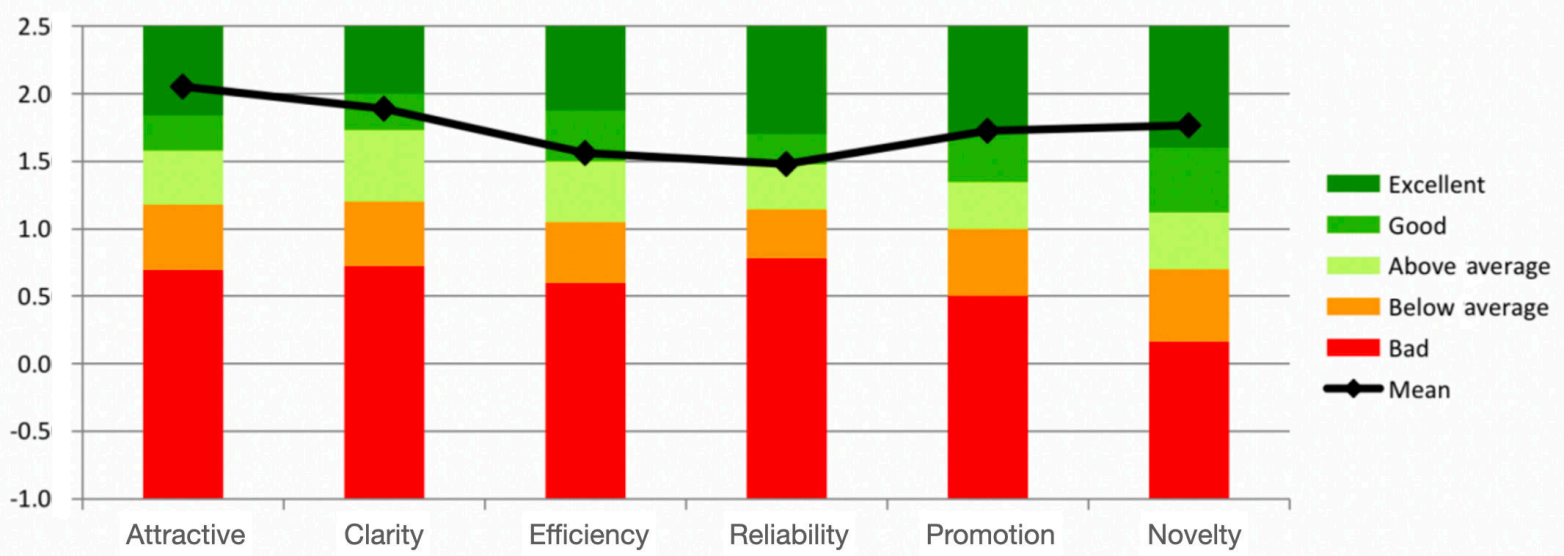
Quality rating score chart.

The scoring results of the 6 dimensions of the UEQ are shown in Table 4. According to the scoring results, it can be visualized that “Attractiveness,” “Clarity” and “Novelty” received high scores (Figure 5). The exercise intervention and functional assessment interface used in this study received the highest score for “attractiveness,” indicating that it really appealed to the patients in the experimental group. The “Clarity” metric, representing the second highest score, suggests that the product presentation is straightforward and easily comprehensible for the user. The elevated score of “Novelty” suggests that the product possesses a commendable level of innovation and captivation, hence enticing customers to engage with it. However, Conversely, “Reliability” receives the lowest rating.

**Table 4.** User Experience Questionnaire score.

Dimension	Mean (SD)		Rating
Attractive	2.05 (0.33)		Excellent
Clarity	1.89 (0.25)		Good
Efficiency	1.56 (0.17)		Good
Reliability	1.48 (0.48)		Above average
Promotion	1.72 (0.2)		Excellent
Novelty	1.76 (0.35)		Excellent

## Discussion

### Principal Findings

The extensive incorporation of digital applications into the rehabilitation process for patients with KOA has been driven by the overarching goal of minimizing the duration of time for them to access specialized rehabilitation experts [5]. More precisely, it is developed based on the thorough enhancement of the widely used framework called “Behavior Change Technique,” as it has a greater propensity to support patients in actively participating in the day-to-day management of their chronic illness [16,25,39]. In addition, the upward trend in the proportion of older individuals who access the Internet demonstrates the potential for internet-based interventions designed to assist this demographic [40]. In addition, studies have yielded inconsistent results concerning the effectiveness of remote rehabilitation for individuals with KOA, primarily attributable to the lack of oversight and guidance [41]. Consequently, a suite of CV-based applications was created to aid patients in the execution of rehabilitation maneuvers with accuracy and to track their advancements through the utilization of noninvasive technology. The results of the study indicated that individuals with KOA who made use of the application reported significantly improved physical function and self-efficacy when compared to the control group.

Research has shown that CV technology–based applications can enhance participants’ physical functioning and alleviate discomfort. This is due to the fact that the capability of integrating behavioral change into digital intervention technologies has been designed to facilitate and encourage adjustments to the patients’ decision-making framework, thereby promoting behavioral change among participants with KOA [42]. The educational aspect of the digital tool encompasses knowledge about the pathology and etiology of osteoarthritis, treatment based on established standards, exercise for osteoarthritis, and methods to alleviate pain and symptoms through adopting healthy behaviors [43,44]. This readily accessible, semisupervised intervention has the potential to be an effective means of alleviating pain and physical dysfunction [39]. Furthermore, our study revealed that the application group experienced a significantly greater improvement in physical functioning compared to the control group. The finding is reminiscent of a study conducted by Tore et al [41], which showed that the quality of physical therapy obtained by telerehabilitation was notably superior to self-management. The difference in quality could perhaps be attributed to the kind and length of the training activities undertaken [45,46]. By means of CV, our application tracks and evaluates the motion of users, thereby facilitating the integration of a customized exercise regimen and aerobic regimen. Contrary to previous research, the investigation did not identify any correlation between the application program and an enhancement in rigidity. Variations in the duration of the studies and the quality and scope of functional support provided may account for this [39]. The reduction in stiffness can be attributed to the fortification of the leg and abdominal muscles; however, a short-term intervention fails to yield substantial outcomes [47,48].

Furthermore, our research revealed that the utilization of a CV-based application resulted in a favorable impact on self-efficacy. Consistent with findings from previous research [11,49,50], digital self-monitoring programs have been associated with relative increases in self-efficacy activation. The promotion of self-management behaviors is facilitated by the provision of information and individualized instruction pertaining to arthritis care, which encourages and assists in the resolution of individual obstacles [51]. Since the factors that influence exercise behavior are motivational and volitional in nature [52,53]. it can be concluded that the application’s monitoring features provided users with the capability to observe their progress and receive prompt feedback on the degree to which their goals were achieved. Conversely, the intervention did not yield any discernible statistically significant effects on negative affect or quality of life. This discovery is consistent with previous inquiries [54], which have shown that digital health interventions have minimal to no effect on adverse emotions and quality of life. Future work should underlie mechanisms responsible for these feelings in patients.

In conclusion, the researchers determined, based on the responses to the user experience questionnaire, that the design principles’ objectives for our application had been effectively met. A majority of the respondents indicated that their encounter with the e-exercise intervention was positive. They believed that the interactive design and real-time feedback enhanced the exercisers’ enjoyment and promoted rehabilitation initiatives. Furthermore, apart from guaranteeing a heightened level of individualized attention, the participants perceived the e-enabled exercise intervention to be more tailored to their rehabilitation needs, thus exceeding apprehensions regarding comfort. The participants articulated that the real-time monitoring effectively captured the motion of the joints, thereby augmenting the efficacy of the measurements. Nevertheless, further investigation is necessary to determine the dependability and accuracy of the sensor-based measurement medium in comparison to the traditional measurement method.

### Limitations

This study has several limitations that warrant discussion. First, the small sample size and the recruitment of participants from communities in close proximity may have led to a concentration of the population distribution, thereby limiting the generalizability of the findings. Second, the pre-experiment revealed that older individuals with insufficient digital health literacy were unable to independently configure and operate the experimental devices. As a result, the experimental protocol was adjusted to involve experimenters facilitating tri-weekly centralized sessions in collaboration with multiple community centers.

While the experimenters’ involvement was limited to device debugging and their interaction with participants was minimal, their presence may have inadvertently influenced the experimental group’s motivation and engagement with the intervention.

The broad age range of 60-80 years is another limitation, as physical capabilities vary significantly within this demographic, potentially introducing variability in the intervention’s outcomes. Future studies should consider stratifying participants into narrower age ranges to enhance sample homogeneity and provide more precise insights into the intervention’s effects across age subgroups.

In addition, the intervention duration was relatively short at 6 weeks, which may not have been sufficient to capture the long-term impact of the application. Future research should extend the study duration, further refine the application design to better accommodate older adults’ needs, and explore tailored strategies to improve digital health literacy. These adjustments would enhance the application’s utility and scalability in diverse community settings, enabling broader adoption and more robust evaluations of its effectiveness.

To address these limitations and enhance future research outcomes, it is recommended to extend the experiment duration, refine the application design to better accommodate older adults’ needs, and explore strategies to improve digital health literacy among the target population. This would enable a more comprehensive evaluation of the application’s capabilities and its potential impact on long-term rehabilitation outcomes.

### Conclusions

The findings of this study demonstrated that the application based on CV technology effectively improved the physical functioning and self-efficacy of participants compared to conventional interventions. This suggests that the application holds promise for replication and implementation within community environments for patients with KOA.

A key novelty of this research is its validation, through a randomized controlled trial, of an application designed according to behavioral change theory principles. The study confirmed that such an application can partially substitute the guidance of a rehabilitation therapist, thereby enhancing exercise rehabilitation outcomes for participants.

In addition, the potential scalability and adaptability of the application in diverse settings warrant further exploration. Its implementation could significantly support older adults in community health management, particularly in addressing the varying levels of digital health literacy among the population. Future research should focus on extending the application’s reach to broader demographics, optimizing its design to better accommodate older adults, and tailoring strategies to improve engagement and usability. This will maximize the application’s practical impact and facilitate its adoption in real-world scenarios.

## Supplementary material

10.2196/63022 Multimedia Appendix 1 Description of the Motion Tracking and Evaluation Algorithm in the CV Application.

10.2196/63022 Checklist 1 CONSORT checklist.

## Abbreviations

ASES: Arthritis Self-Efficacy Scale
CONSORT: Consolidated Standards of Reporting Trials
CV: computer vision
GDS: Geriatric Depression Scale
KOA: knee osteoarthritis
NRS: numeric rating scale
ROM: Range of motion
UEQ: User Experience Questionnaire
WOMAC: Western Ontario and McMaster Universities Arthritis Index
