# Supplementary material for: Effects of a Computer Vision–Based Exercise Application for People With Knee Osteoarthritis: Randomized Controlled Trial
Source: JMIR Mhealth Uhealth. 2025 May 12;13:e63022. doi: 10.2196/63022 (PMC12088618; doi:10.2196/63022)
Supplement: Multimedia Appendix 1 [file mhealth-v13-e63022-s001.docx]

Appendix File: Description of the Motion Tracking and Evaluation Algorithm in the CV Application


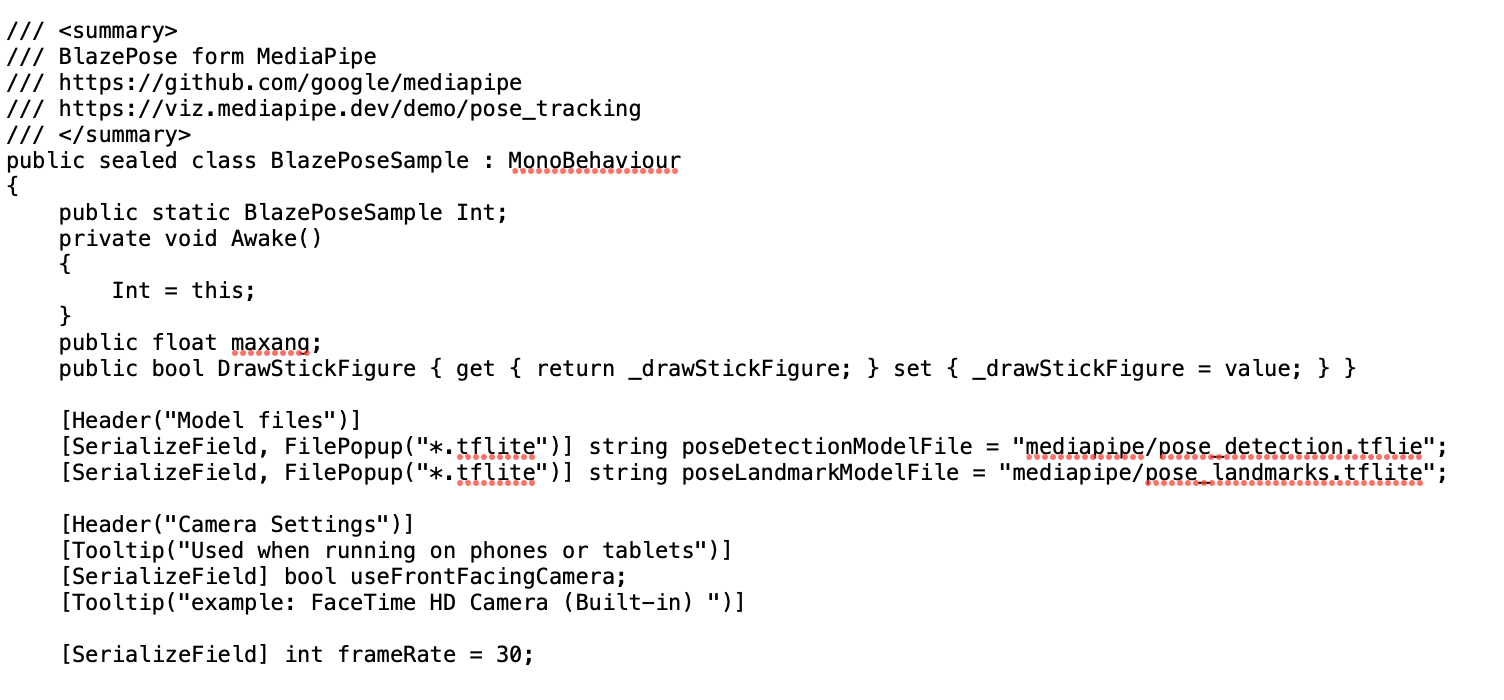


Figure 1 Partial code

This study uses a computer vision algorithm based on MediaPipe BlazePose for motion tracking and evaluation. The following is a detailed description of the technical implementation:

1.Keypoint Detection Model Two TensorFlow Lite models are used:

pose_detection.tflite: Detects the approximate position of the body and generates bounding boxes.

pose_landmarks.tflite: Based on the bounding box, generates precise coordinates for 33 keypoints of the body, including joints, eyes, ears, mouth, and other important positions.

2.Pose Keypoint Tracking

Each keypoint is described by its (x, y, z) coordinates and the visibility value (confidence level). In each frame, the system calculates bone connections (e.g., joint angles) using the keypoints and applies them for real-time motion evaluation.

3.Real-Time Motion Capture and Visualization

The system captures human movements in real-time via a camera and uses the models mentioned above to identify keypoints. World Coordinate System Processing: The system performs matrix transformations on the keypoint coordinates, adjusting them to the world coordinate system from the camera's perspective for subsequent pose evaluation and visualization.

4.Motion Standard Analysis

Motion Angle Calculation: The system calculates joint angles through vector dot products between skeletal keypoints and compares them with predefined standard value ranges (e.g., knee flexion/extension angle between 25°-35°).

Motion Classification: Based on the angle and changes in keypoint positions, the system assesses the degree of standardization of the movement and provides real-time feedback (e.g., "Standard" or "Non-standard").

5.Risk Monitoring

The system includes a feature for detecting risky movements (e.g., falls or excessive joint bending), triggering an alert sound, and pausing motion capture.

6.Data Filtering and Optimization

A velocity filter (FilterVelocityScale) is introduced to smooth the time-series data of keypoint positions, reducing the impact of noise on keypoint tracking.


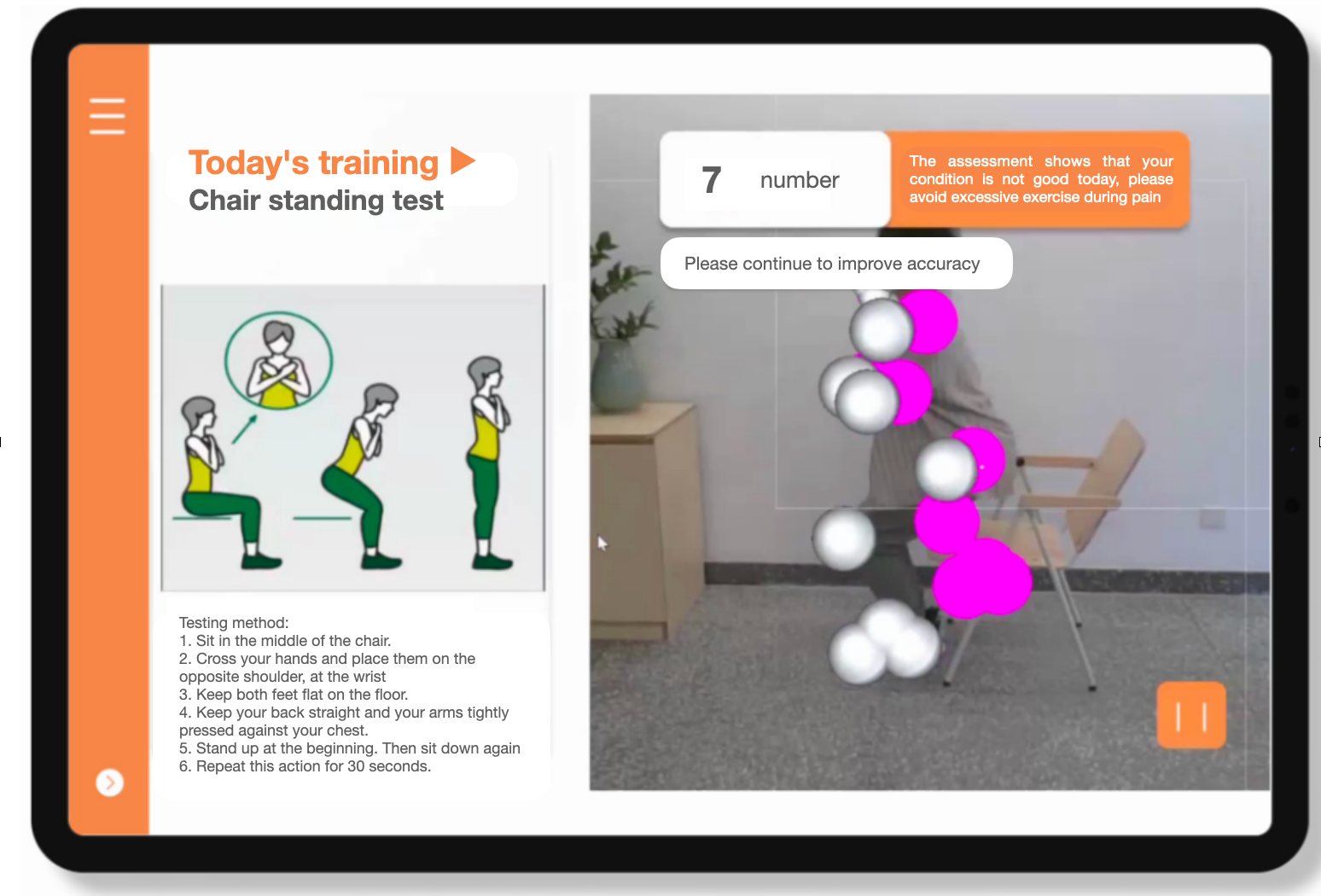

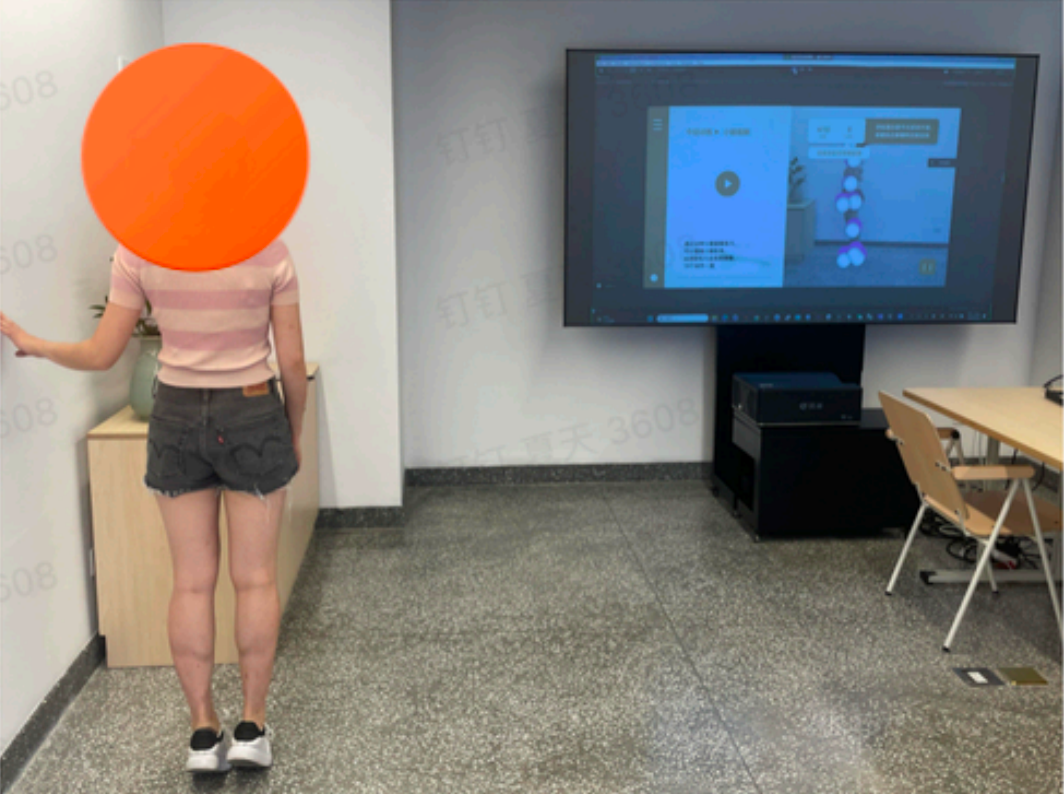


This is an illustration of the participant's motion process in computer vision. The white and purple spheres represent various joint keypoints (we have enlarged the size for clarity, but this will not be displayed in the actual product). The product tracks the completion of movements by calculating the movement of these points.
